# Improving shared decision-making about cancer treatment through design-based data-driven decision-support tools and redesigning care paths: an overview of the 4D PICTURE project

**DOI:** 10.1177/26323524231225249

**Published:** 2024-02-12

**Authors:** Judith A. C. Rietjens, Ingeborg Griffioen, Jorge Sierra-Pérez, Gaby Sroczynski, Uwe Siebert, Alena Buyx, Barbara Peric, Inge Marie Svane, Jasper B. P. Brands, Karina D. Steffensen, Carlos Romero Piqueras, Elham Hedayati, Maria M. Karsten, Norbert Couespel, Canan Akoglu, Roberto Pazo-Cid, Paul Rayson, Hester F. Lingsma, Maartje H. N. Schermer, Ewout W. Steyerberg, Sheila A. Payne, Ida J. Korfage, Anne M. Stiggelbout

**Affiliations:** Department of Public Health, Erasmus MC, University Medical Center Rotterdam, PO Box 2040, Rotterdam 3000 CA, The Netherlands; Department of Design, Organisation and Strategy, Faculty of Industrial Design Engineering, Delft University of Technology, Delft, The Netherlands; Department of Design, Organisation and Strategy, Faculty of Industrial Design Engineering, Delft University of Technology, Delft, The Netherlands; Medical Decision Making, Department of Biomedical Data Sciences, Leiden University Medical Center, Leiden, The Netherlands; Department of Engineering Design and Manufacturing, University of Zaragoza, Zaragoza, Spain; Institute of Public Health, Medical Decision Making and Health Technology Assessment, Department of Public Health, Health Services Research and Health Technology Assessment, UMIT TIROL – University for Health Sciences and Technology, Hall in Tirol, Austria; Institute of Public Health, Medical Decision Making and Health Technology Assessment, Department of Public Health, Health Services Research and Health Technology Assessment, UMIT TIROL – University for Health Sciences and Technology, Hall in Tirol, Austria; Institute for History and Ethics of Medicine, Technical University of Munich, Munich, Germany; Institute of Oncology Ljubljana, Medical Faculty Ljubljana, University of Ljubljana, Ljubljana, Slovenia; Department of Oncology, National Center for Cancer Immune Therapy, Herlev, Denmark; Panton, Deventer, The Netherlands; Center for Shared Decision Making, Vejle/Lillebaelt University Hospital of Southern Denmark, Vejle, Denmark; Institute of Regional Health Research, Faculty of Health Sciences, University of Southern Denmark, Odense, Denmark; Department of Design and Manufacturing Engineering, University of Zaragoza, Zaragoza, Spain Fractal Strategy, Zaragoza, Spain; Department of Oncology–Pathology, Karolinska Institute, Stockholm, Sweden; Breast Cancer Centre, Cancer Theme, Karolinska University Hospital, Karolinska CCC, Stockholm, Sweden; Department of Gynecology with Breast Center, Charité Universitätsmedizin Berlin, Berlin, Germany; European Cancer Organisation, Brussels, Belgium; Lab for Social Design, Design School Kolding, Kolding, Denmark; Department of Medical Oncology, Instituto de Investigación Sanitaria de Aragón, Hospital Universitario Miguel Servet, Zaragoza, Spain; School of Computing and Communications, University Centre for Computer Corpus Research on Language, Lancaster University, Lancaster, UK; Department of Public Health, Erasmus MC, University Medical Center Rotterdam, Rotterdam, The Netherlands; Department of Medical Ethics and Philosophy of Medicine, Erasmus MC, University Medical Center Rotterdam, Rotterdam, The Netherlands; Department of Public Health, Erasmus MC, University Medical Center Rotterdam, Rotterdam, The Netherlands; Medical Decision Making, Department of Biomedical Data Sciences, Leiden University Medical Center, Leiden, The Netherlands; International Observatory on End of Life Care, Lancaster University, Lancaster, UK; Department of Public Health, Erasmus MC, University Medical Center Rotterdam, Rotterdam, The Netherlands; Medical Decision Making, Department of Biomedical Data Sciences, Leiden University Medical Center, Leiden, The Netherlands Erasmus School of Health Policy and Management, Erasmus University, Rotterdam, The Netherlands; 1 Department of Public Health, Erasmus MC, University Medical Center Rotterdam, Rotterdam, The Netherlands; 2 Department of Design, Organisation and Strategy, Faculty of Industrial Design Engineering, Delft University of Technology, Delft, The Netherlands; 3 Medical Decision Making, Department of Biomedical Data Sciences, Leiden University Medical Center, Leiden, The Netherlands; 4 Department of Engineering Design and Manufacturing, University of Zaragoza, Zaragoza, Spain; 5 Institute of Public Health, Medical Decision Making and Health Technology Assessment; Department of Public Health, Health Services Research and Health Technology Assessment, UMIT TIROL – University for Health Sciences and Technology, Hall in Tirol, Austria; 7 Institute of Oncology Ljubljana, Medical faculty Ljubljana, University of Ljubljana, Ljubljana, Slovenia; 8 Department of Oncology, National Center for Cancer Immune Therapy, Herlev, Denmark; 10 Center for Shared Decision Making, Vejle/Lillebaelt University Hospital of Southern Denmark, Vejle, Denmark; 15 Breast Cancer Centre, Cancer Theme, Karolinska University Hospital; Karolinska CCC, Stockholm, Sweden; 16 Department of Gynecology with Breast Center, Charité Universitätsmedizin Berlin, Berlin, Germany; 17 European Cancer Organisation, Brussels, Belgium; 18 Lab for Social Design, Design School Kolding, Kolding, Denmark; 21 Department of Medical Ethics and Philosophy of Medicine, Erasmus MC, University Medical Center Rotterdam, Rotterdam, The Netherlands; 24 Department of Linguistics and English Language, Lancaster University, Lancaster, United Kingdom; 25 Department of Urology, Cancer Institute, Erasmus MC, University Medical Center Rotterdam, Rotterdam, The Netherlands; 26 Leiden Institute of Advanced Computer Science, Leiden University, Leiden, The Netherlands; 27 Department of Medical Informatics, Erasmus MC, University Medical Center Rotterdam, Rotterdam, The Netherlands; 28 Department of Surgery, Cancer Institute, Erasmus MC, University Medical Center Rotterdam, Rotterdam, The Netherlands; 29 Department of Palliative Care, Lancaster Medical School, Lancaster, UK; 30 Marie Curie Hospice Liverpool, Liverpool University Hospitals NHS Foundation Trust, Liverpool, UK; 31 NABON Breast Cancer Audit (NBCA), Dutch institute of Clinical Auditing (DICA); 32 Department of Computer Science and Systems Engineering, University of Zaragoza, Zaragoza, Spain; 33 Department of Medical Oncology, Miguel Servet University Hospital, Facultad de Medicina. Universidad de Zaragoza, Instituto Investigación Sanitaria Aragón, Zaragoza, Spain; 34 Dutch Institute for Clinical Auditing, Leiden, The Netherlands; 35 Department of Surgery, Leiden University Medical Center, Leiden, The Netherlands; 36 Department of Medical Oncology, Leiden University Medical Center, Leiden, The Netherlands; 37 Department of Information Technology & Digital Innovation, Leiden University Medical Center (LUMC), Leiden, The Netherlands; 38 Department of Radiation Oncology (MAASTRO), GROW School of Oncology and Developmental Biology (GROW), Maastricht University Medical Center, Maastricht, The Netherlands

**Keywords:** artificial intelligence, care paths, communication, cost-effectiveness, design, ethics, prognostic modelling, public health, shared decision-making

## Abstract

**Background:**

Patients with cancer often have to make complex decisions about treatment, with the options varying in risk profiles and effects on survival and quality of life. Moreover, inefficient care paths make it hard for patients to participate in shared decision-making. Data-driven decision-support tools have the potential to empower patients, support personalized care, improve health outcomes and promote health equity. However, decision-support tools currently seldom consider quality of life or individual preferences, and their use in clinical practice remains limited, partly because they are not well integrated in patients’ care paths.

**Aim and objectives:**

The central aim of the 4D PICTURE project is to redesign patients’ care paths and develop and integrate evidence-based decision-support tools to improve decision-making processes in cancer care delivery. This article presents an overview of this international, interdisciplinary project.

**Design, methods and analysis:**

In co-creation with patients and other stakeholders, we will develop data-driven decision-support tools for patients with breast cancer, prostate cancer and melanoma. We will support treatment decisions by using large, high-quality datasets with state-of-the-art prognostic algorithms. We will further develop a conversation tool, the Metaphor Menu, using text mining combined with citizen science techniques and linguistics, incorporating large datasets of patient experiences, values and preferences. We will further develop a promising methodology, MetroMapping, to redesign care paths. We will evaluate MetroMapping and these integrated decision-support tools, and ensure their sustainability using the *Nonadoption, Abandonment, Scale-Up, Spread, and Sustainability* (NASSS) framework. We will explore the generalizability of MetroMapping and the decision-support tools for other types of cancer and across other EU member states.

**Ethics:**

Through an embedded ethics approach, we will address social and ethical issues.

**Discussion:**

Improved care paths integrating comprehensive decision-support tools have the potential to empower patients, their significant others and healthcare providers in decision-making and improve outcomes. This project will strengthen health care at the system level by improving its resilience and efficiency.

## Plain language summary

Improving the cancer patient journey and respecting personal preferences: an overview of the 4D PICTURE project

The 4D PICTURE project aims to help cancer patients, their families and healthcare providers better undertstand their options. It supports their treatment and care choices, at each stage of disease, by drawing on large amounts of evidence from different types of European data. The project involves experts from many different specialist areas who are based in nine European countries. The overall aim is to improve the cancer patient journey and ensure personal preferences are respected.

## Background

Treatment decision-making by patients with cancer, their significant others and clinicians can be complex,^1^ in particular when a choice has to be made between different treatment regimens (such as chemotherapy, surgery, radiotherapy – or no treatment) with different risk profiles, and effects on survival and quality of life. This pertains to curative oncological care as well as palliative care. Decision-support tools, which are computer-based tools developed to support decision analysis and participatory processes, have the potential to lead to improved access to innovative, high quality, oncological care. They could enhance patient empowerment and treatment adherence, leading to better health outcomes and more health equity.^2^ The latter is of the essence, as access to the latest state-of-the art care is now often easier for privileged groups, and differs between countries.^3^ Moreover, underrepresentation in medical research of underserved groups such as older persons, migrants and persons with multiple conditions, is leading to bias and less optimal care for those groups.^4^ A plethora of decision-support tools for clinical decision-making in oncology have been developed, including some specifically to be used by patients and clinicians together. However, many fail to live up to their expectations. To reach the full potential of decision-support tools in oncological care, three main challenges need to be overcome, related to quality, patient preferences and implementation. In the 4D PICTURE project, we will address these in a comprehensive manner.

### Challenge 1: The quality and outcomes of decision-support tools are insufficient

Current decision-support tools have various limitations. First, prognostic algorithms are often developed on small single-study datasets using poor methodology.^5^ This not only limits their application to the diverse patient population seen in a specific clinical practice, but also their generalizability to other settings. This holds even more true for models predicting treatment benefits, which require larger sample sizes and assumptions. Importantly, invalid models might lead to harmful decisions due to wrong predictions. Second, to achieve high-quality oncological care, predicting clinical outcomes such as survival are only one piece of the puzzle. Specifically, in the context of increasing survival rates, prediction of patient reported outcomes (PROMS) such as quality of life are also key. Information on these outcomes is important in supporting patients in navigating preference-sensitive treatment decisions, in which there is not one best treatment but decisions depend on patients’ values and preferences for these outcomes. Models predicting such outcomes are rare. In the 4D PICTURE project, we will develop these models, focusing on three types of cancers that are well known for such complex preference-sensitive treatment: melanoma, prostate cancer and breast cancer. Third, each prediction carries inherent uncertainty. At present, uncertainty is not explicitly considered or communicated with patients, while the uncertainty surrounding the predicted outcomes of different treatments clearly will affect decisions. This is also relevant for policymakers, as prognostic models are a key opportunity to deliver care that is more appropriate and to improve value by limiting overuse of costly resources, and direct these resources towards high-risk patients.^6^ This will also be addressed in the project.

### Challenge 2: Decision-support tools do not sufficiently address patients’ preferences

Every patient is different, and no cancer is the same. Personalized medicine catered for the smart combination of health data and new technologies, has the potential to change patients’ prognosis. However, when preference-sensitive treatment decisions need to be made, individual predicted outcomes should be weighed against patients’ preferences and needs in the context of their personal situation, in every phase of the disease. Oncological prognostic models reinforce a ‘tumourized’ approach of decision-making (as they do not include patients’ treatment preferences), rather than a person-centred approach. Shared decision-making (SDM) is such a person-centred approach. It is increasingly advocated as the preferred decision-making model for preference-sensitive decisions, to include both the best available evidence as well as patients’ preferences in decision-making.^7^ SDM is one of the 10 specific rights of the European Code for Cancer Patients. At its core, SDM is a process in which decisions are made in a collaborative way between clinicians and patients and their significant others, based on trustworthy information in accessible formats.^8^ Research has shown that SDM results in increased satisfaction (of both patients and professionals) with the communication and the decision, more informed decisions,^9,10^ and better coping with side effects.^11^ Patients who have been supported in SDM by a decision aid tend to choose more conservative, less extensive treatment options.^12^ This means that SDM could also lead to lower societal financial burden, more equitable distribution of resources and more sustainable health care.

Incorporating patients’ preferences in decision-making is a difficult and complex task for clinicians. It is not always clear which aspects are important for patients (e.g. the burden of treatment, treatment focusing on comfort or on living as long as possible, or of side effects). Critically, clinicians do not always ‘speak the language of patients’. Several tools have been developed to include patients’ preferences in medical decision-making. Most of these tools are aimed at supporting patients to express preferences about a fixed set of treatment options. Unfortunately, their effectiveness is limited, and many tools are difficult to understand for vulnerable patients with limited health literacy.^13^ Barriers to sensitive conversations about patients’ preferences for treatment and care are ingrained in the healthcare system: a power imbalance between clinicians and patients,^13^ reluctance to engage in personal conversations and a taboo to talk about cancer, serious illness or palliative care.^14^

What is needed is a radical new type of conversation tool, one that invites clinicians, cancer patients and their wider support system to engage in meaningful conversations about the ‘lived experience’ of cancer. One that can accommodate and appreciate the different ways in which patients experience and navigate their illness. It is such a tool that we will develop, using the ‘largest and least utilized resource in healthcare’: the patient.^15^ Increasingly, patients share their experiences and knowledge about their conditions through first-person accounts on blogs, social media and online fora. The main advantage of such data is that it offers an uncensored and thus a potentially wider pool of information unrestricted by the medical setting whose volume is not easily obtainable by other means. Moreover, patients are more likely to share some types of information with fellow patients than with their physicians,^16^ hence providing access to authentic accounts of patients’ experiences, challenges and preferences. However, one limitation to this is that we can expect a bias as to who has access to the online space, and also a bias in who shares their experiences in the online world.^16^ The systematic textual analysis of data will provide a rich source to support the development of conversation tools, one that builds on their own language, metaphors and narratives, as exemplified below in section ‘Methods’.

### Challenge 3: Implementation of decision-support tools is fragmented and uncoordinated

Decision-support tools often fail to be implemented successfully after the lifetime of a project, and even successfully implemented decision-support tools fail to be sustained over time.^17^ Decision-support tools are not always adopted by clinicians or patients due to time constraints in the clinical encounter, the difficulties for patients to understand them, the perceived negative impact of the use of decision-support tools on the doctor–patient relationship and lack of fit into the care path.^18–22^ Some oncologists indicate that they merely consult decision-support tools without patients, before the consultation, to support their own decision-making or to convince patients. Yet there is some evidence that patients may consider decision-support tools helpful when making treatment decisions.^23^ In our recent research, clinicians, patients and their significant others described that inefficient care paths made it hard for them to participate in SDM.^1^ Inefficient care paths included logistic problems (such as waiting times for tests and unavailability of information from referring hospitals), lack of overview of the entire treatment trajectory (including unclear responsibilities) and poor and inconsistent information provision. This may lead to high levels of stress, fear and disempowerment of patients and their significant others.^1^ Therefore, sustainable implementation of decision-support tools requires a wider systems approach, focusing not solely on the medical encounter but on the larger care path, appreciating the longitudinal nature of decisions that require ongoing adherence. Each consultation should not be considered as stand-alone, but as a ‘chapter in the entire story of a person’s illness’.^1^

In the 4D PICTURE project, we will redesign patients’ care paths, leveraging untapped potential of design methodologies, to better accommodate decision-support tools and support SDM. We will use service design methodology, following an established design process^24^ and a design thinking approach^25^: an iterative process including further specifying the problems to be solved and, through co-design with stakeholders, generating proposals to solve these problems. Service design has been applied successfully in several other healthcare improvement initiatives, however, its application in oncology is limited and its use to support SDM is novel. We will build on the novel service design methodology MetroMapping,^26^ which will be further explained in section ‘Design, methods and analysis’.

In this article, we provide an overview of the 4D PICTURE project, including its work packages, methods and foundations. It is meant to highlight how this 5-year international project, with teams in Austria, Belgium, Denmark, Germany, the Netherlands, Slovenia, Spain, Sweden and the UK, will address the above challenges. Moreover, it will show that for such challenges, interdisciplinary research is needed, including service design, data science, ethics, the social sciences and health economy, and involving clinicians from various medical specialties and patient representatives. Various designs and methods are used in the different work packages, which are discussed below.

## Aim and Objectives

The central aim of the 4D PICTURE project is to redesign patients’ care paths and develop and integrate evidence-based decision-support tools to improve decision-making processes in cancer care delivery.

This central aim translates into the following specific scientific objectives:

Objective 1. To develop data-driven algorithms, resulting in prognostic tools, accompanied by indications of certainty of estimates of individual benefit and harms from treatment, and supported by a structure for FAIR data management and dynamic analysis (work package 2: Modelling).
Objective 2. To develop a conversation tool for cancer patients, their significant others, their clinicians and citizens, based on text mining analyses of patient experience ‘big’ data and citizen science methods (work package 3: Text mining).
Objective 3. To further develop and apply internationally a promising service design methodology, called MetroMapping, to redesign care paths (work package 4: Design).
Objective 4. To evaluate the service design methodology ‘MetroMapping’ and the decision-support tools. Additionally, to systematically evaluate the short- and long-term costs and effects of MetroMapping methodology (work package 5: Practice).
Objective 5. To guide policy-making by exploring the generalizability of the MetroMapping methodology and the decision-support tools for patients with other types of cancer and in other countries (work package 6: Policy).
Objective 6. To guarantee the development of ethically and socially responsible decision-support tools by integrating social and ethical considerations in the process (work package 7: Ethics).

## Design, methods and analysis

In this section, we first address some unique aspects of our project, discuss the framework that we use for the development, evaluation and implementation of our tools and methods and provide a generic overview of the various methods we use. This is followed by the studies that will be carried out in the different work packages.

### Unique aspects of the 4D PICTURE project

#### Interdisciplinary and international approach

Research on decision-making in oncology is typically conducted in silos with little crossover between technical data sciences, patient preferences science and implementation science. This impedes progress. To identify innovative solutions that can tackle profound challenges facing the field of data-driven decision-support tools, we must move beyond data, information and technology towards sociotechnical systems that recognize both people and technologies and their interdependencies within the organizational context. We will therefore take an interdisciplinary, multi-stakeholder approach in this project, to truly integrate health services research, oncology, palliative care, data science, design, citizen science, ethics, health economics and implementation science to support more equitable, innovative and sustainable healthcare systems. The 4D PICTURE project team is based in nine countries: Austria, Belgium, Denmark, Germany, the Netherlands, Slovenia, Spain, Sweden and the UK.

#### Patient and public involvement

Involvement of patients, patient representatives and citizens, often referred to as patient and public involvement (PPI), is at the heart of the 4D PICTURE project. Co-production of the knowledge and products generated in the 4D PICTURE project will contribute to the quality, feasibility and eventual impact of the project. It is a sharing of power, with researchers and PPI members working together to develop the agenda, design and implement the research, and interpret, disseminate and implement the findings.^27^

PPI in the 4D PICTURE project will be undertaken as follows:

- We will install a Patient and Public involvement Board: in each country, three to five cancer patients, family caregivers and/or their representatives. They will provide regular advice about key issues in the work packages.
- We have prominent patient representation among the project partners (ECO) and Advisory Board members (Europa Donna, Europa Uomo).
- Some of the consortium members are (ex) cancer patients themselves and can draw from their personal experiences.

#### Methodological framework for development, evaluation and implementation of healthcare applications

The conceptual basis of the 4D PICTURE project is guided by the evidence-based, theory-informed NASSS conceptual framework that guides development, evaluation and implementation of data and technology applications in healthcare.^21^ This framework will support our understanding of how to develop and implement sustainable decision-support tools. This matters because about 80% of data and technology projects in healthcare fail to be implemented successfully after the lifetime of a project.^17^ The underlying idea of the NASSS model is that failure of sustainable implementation is due to high levels of complexity in one or more of seven domains (micro, meso, macro levels). These domains relate to (1) the illness or condition, (2) the technology of the tool, (3) the value proposition – both supply and demand side, (4) the adopter system: clinicians, patients and their significant others, (5) the organization, (6) the wider system, including the context of policy, regulatory or professional bodies and public perceptions and (7) embedding and adapting over time. Complexities may relate to, for instance, unpredictable disease trajectories, (costs of) software or lack of involvement of stakeholders. For sustainable implementation of the decision-support tools as developed in the 4D PICTURE project, we will identify and lower complexity in these seven domains.^22^

#### Multiple methods approach

In the development of the decision-support tools, as well as a balanced evaluation of their value for cancer patients, their significant others, clinicians, healthcare systems and the larger society, we integrate:

- Various research designs, including qualitative approaches such as interviews, focus groups and a Delphi study, as well as quantitative methodology, such as prognostic modelling, text mining and an evaluation study with observations of real-world implementation of the developed tools.
- Various data collection methods, including questionnaires, interviews, audiotaped consultations and unstructured patient data from patient fora, social media and blogs.
- Various analysis techniques, including quantitative statistics, qualitative coding and automated machine learning.

#### The MetroMapping methodology and integration of the decision-support tools

**Figure 1.**
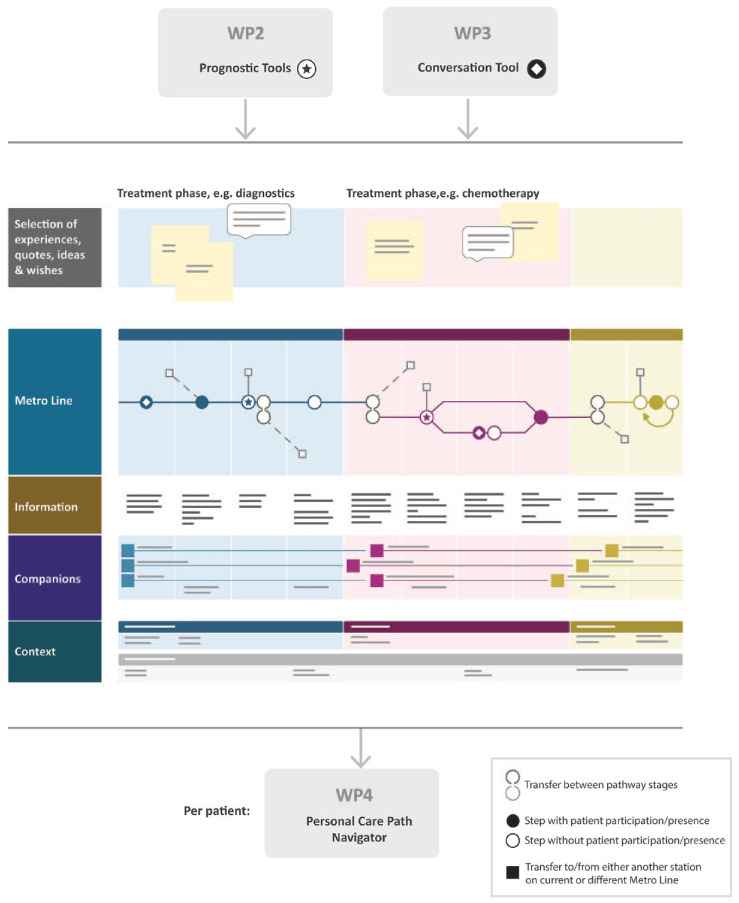
The MetroMapping methodology.

We will further develop a promising service design methodology, called MetroMapping. MetroMapping is a methodology through which patients’ care paths can be optimized and redesigned. It uses service design methodology, which aims to create a valuable ‘service’ through a human-centred, holistic and iterative approach acceptance. The MetroMapping methodology involves five layers (see Figure 1):

- Layer Experience: All experiences with the current care path are assessed from patients, their significant others and clinicians. We use the novel methods from work package 3 (Text mining) and standard design probe methods.
- Layer Metro line: a visual overview of the entire care pathway and decision moments. Each phase in the diagnosis and treatment pathway is represented in colour, and transitions from one branch (e.g. the diagnosis process) to another (e.g. chemotherapy or radiotherapy) are shown. This visualizes the choices and what they depend on (e.g. the result of a scan or a patient’s preference) and the moments a patient has contact with healthcare providers.
- Layer Information: Patients need information, for example to prepare for a decision, to know the place and location of treatments or to be prepared for side effects. This layer clarifies the information needed and in what way it can be provided (e.g. in a conversation, with a website or app). By aligning the information layer with the metro line, it becomes clear that with a change in the trajectory new information may be needed.
- Layer Companions: Providing insight into who (and in what role) accompany the patient during the care trajectory: all people who ensure that the patient gets the desired and medically best treatment, adheres to treatment and receives care quickly in an acute situation. Important roles are those of the significant other, the treating physician, but also of the nurse or case manager.
- Layer Context: Concerning the physical context in which the trajectory takes place and the artefacts (physical objects) that patients may encounter. The physical context may be at home, or in, for example, a primary care practice or a hospital. One can think of consultation rooms, but even of parking garages. Which route do patients, their families and clinicians follow, and how are they influenced by what they see or experience? Artefacts may concern devices patients use, for example, a port-a-cath for the administration of chemotherapy.

Once experiences with the current, inefficient care path have been assessed and the trajectory has been visualized (an effort including patients, their significant others, clinicians and quality of care staff), it can subsequently be redesigned to improve the healthcare delivery decision-making processes. Important assets of the MetroMapping methodology are its flexibility for heterogeneous cancer care paths and its intuitively attractive visual language, enabling both engagement of patients with various levels of health literacy and multidisciplinary collaboration.

In the information layer, prognostic tools and a conversation tool will be integrated (see Figure 1).

Prognostic tools are tools that help show – with a carefully designed user-centred interface – what outcomes may be expected given current care, or how cancer treatments might affect survival rates and quality of life of cancer patients. Prognostic tools are based on robust and transparent modelling of structured clinical data. They will be embedded in a website.

A conversation tool, with visual and textual components, aims to open up conversations about cancer (including preferred language and personal preferences). Its development is based on methods combining text mining and citizen science, using unstructured patient experience data, such as expressed in blogs and patient fora.

Aiming to maximize the potential implementation of MetroMapping methodology in different cultures and settings across Europe, MetroMapping can also be used without these two decision-support tools, depending on local circumstances and needs. Also, each of these tools can be used as a stand-alone tool.

**Figure 2.**
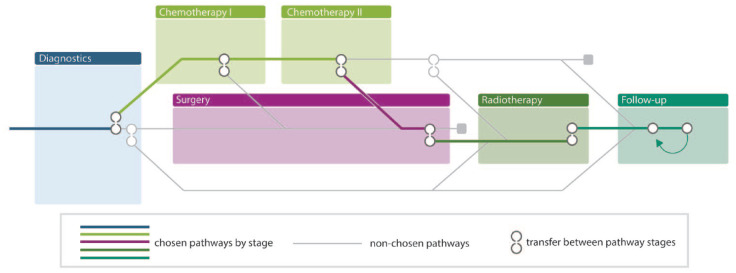
The Personal Care Path Navigator.

After the MetroMapping process has been completed, an individual patient tool called Personal Care Path Navigator can be created (see Figure 2). This tool results from the redesigned care path and details the *individual* care trajectory of a patient. It is a personal diagnostic and treatment plan (trajectory), collaboratively created by a patient, his or her significant other, and clinician in a process of SDM, that fits the needs and preferences of that patient. The tool includes, for instance, timing and number of check-ups, appointments, involved clinicians and roles. The tool is adaptive, for example, when a specific treatment is not effective.

The prognostic tools will be developed in work package 2 (Modelling), the conversation tool in work package 3 (Text mining) and the Personal Care Path Navigator in work package 4 (Design); the work packages are all described in the next section.

### The work packages

**Figure 3.**
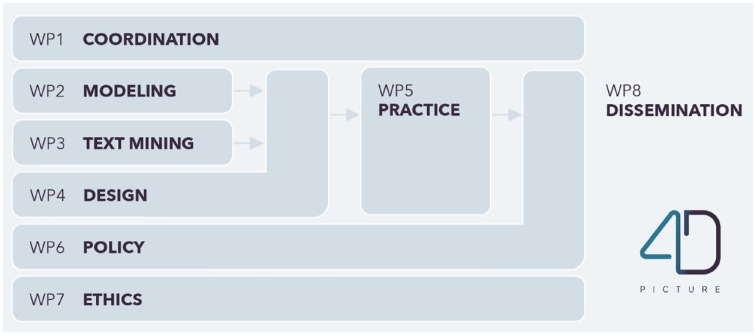
The work packages of the 4D PICTURE project.

In the next sections, we will describe our proposed methods per objective and work package. The Supplemental Appendix provides an overview of the related deliverables. Figure 3 visualizes the interconnectedness between the work packages. Work package 1 (Coordination) concerns the coordination of the project and is left out of the descriptions.

#### Work package 2: Modelling (Objective 1, Months 1–54)

The overall objective of work package 2 Modelling is to support decision-making about cancer treatment by better predicting outcomes with and without a specific treatment for patients with breast cancer, prostate cancer or melanoma. We have ensured access to a unique combination of high-quality datasets, also including data from disadvantaged populations, from various sources, containing data on ~17,000 melanoma patients, ~186,000 breast cancer patients and ~34,000 prostate cancer patients which will be managed and analysed according to FAIR data principles. The datasets contain a rich set of predictors, including clinical, tumour, imaging and biomarker data as well as patient and treatment characteristics. In addition to clinical outcomes, many datasets also contain PROMS (i.e. quality of life measures). In collaboration with work package 7 (Ethics), we will report on the representativeness of the data sets.

To predict outcomes, we will first apply and expand on methods to reliably produce predictions for individual patients (prognostic modelling). To inform treatment decisions the goal is not only to predict outcomes, but to quantify the expected benefits of specific treatments, which differ by patient. Such heterogeneity of treatment effect will be analysed from the framework set out in the Predictive Approaches to Treatment effect Heterogeneity (PATH) Statement.^28^ We aim to develop at least six algorithms. Subsequently, we will address causes and consequences of uncertainty in prognostic algorithms and assess possibilities for presentation of uncertainty to policymakers, clinicians, patients and citizens, with work package 6 (Policy). The prognostic algorithms will be developed into a decision-support tool, for instance, through implementation into a digital calculator, and integrated in the MetroMapping methodology in work package 4 (Design).

#### Work package 3: Text mining (Objective 2, Months 1–54)

The objective of work package 3 (Text mining) is both to develop a conversation tool and to obtain input for care path redesign for patients with cancer, their significant others, their clinicians and citizens based on text mining analyses of patient experience ‘big data’ and citizen science methods. The conversation tool will be based on the ‘Metaphor Menu for People Living with Cancer’, a resource for patients developed by Lancaster team members as part of a previous project (http://wp.lancs.ac.uk/melc/the-metaphor-menu/), which will be extended to three new languages (Danish, Dutch and Spanish). These will feed into the MetroMapping methodology in work package 4 (Design) and will be evaluated *via* interviews and questionnaires as part of work package 5 (Practice). Metaphor and narratives are well known to be framing devices that people use both to express and make sense of their experiences. As such, metaphors can provide insights into people’s views, challenges and attitudes and can be used in the conversations between clinicians and patients and their significant others. The analysis will be used to produce exemplar metaphors and narratives that capture particular critical moments in patients’ cancer experiences over time (e.g. diagnosis, the start of treatment, life after treatment, palliative care and end-of-life decisions). We will apply an interdisciplinary approach that combines the strengths of text mining or natural language processing techniques, corpus linguistics and qualitative (narrative) research to efficiently convert the stories of experiences of people with cancer and their significant others into usable knowledge about how they experience their care trajectory, including palliative care. The tool will be integrated in the MetroMapping methodology in work package 4 (Design).

#### Work package 4: Design (Objective 3, Months 6–36)

The objective of this work package is to further develop and test internationally the service design methodology MetroMapping (https://metromapping.org/en/) to redesign care paths and to integrate the prognostic tools and the conversational tool as developed in work package 2 (Modelling) and work package 3 (Text mining). Service design methodology is characterized by its holistic nature, sequential and iterative application, application in the real context and ability to understand the influence of contextual factors (such as fit of the tool into the clinical pathway). A key element is co-design with important stakeholders, in this project to be understood as co-designing with patients and their significant others as service users, and clinicians and quality of care staff as service providers.

In this work package, MetroMapping will be executed by local service designers in hospitals in three countries in which forerunners use service design in oncology (West: Netherlands, South: Spain, North: Denmark). Per country, the local team will select two care paths (breast, prostate cancer or melanoma), including palliative care, based on the local situation. The redesign of the care path will be done by means of service design tools to detect intangible aspects of the experience of services, such as stakeholders’ preferences and needs and context elements that can affect the decision-making. The design tools will be used in context, during actual interactions between stakeholders, and help to identify the existing (or lacking) information and touch points. We will develop MetroMapping further, including guidelines and strategies for its implementation in an international context.

#### Work package 5: Practice (Objective 4, Months 12–56)

This work package concerns the empirical evaluation of MetroMapping and the decision-support tools. The adapted service design methodology MetroMapping and decision-support tools developed in the 4D PICTURE project will be applied in a multi-centre mixed methods study (Denmark, the Netherlands and Spain) to evaluate (1) the use of MetroMapping in routine practice, (2) the developed decision-support tools (i.e. the prognostic and conversation tools), (3) the use of the conversation tool among citizens and (4) the short- and long-term effects, costs and cost-effectiveness of MetroMapping. A mixed methods approach using quantitative and qualitative measures will be applied and results will be reported based on method-specific standard guidelines.

Ad (1): The use of MetroMapping in routine practice will be evaluated using a pre-test–post-test design. We evaluate the impact of the implementation of MetroMapping on the decision-making process and other outcomes using different questionnaires, such as the I-SHARE for SDM and the Decisional Regret Scale.^29–32^ Uni- and multivariate statistics will be used to compare pre-/post-test outcome changes, controlling for socio-demographic and clinical characteristics. To detect a small-to-medium effect (Cohen’s *d* of 0.3), with a power of 0.80 and an alpha of 0.05, we need a sample size of 176 patients both in the pre- and post-implementation phase (*N* = 352). We increase the sample size to 500 (100 breast, 100 prostate, 50 melanoma, in both the pre- and post-implementation phase) to account for the unknown heterogeneity across countries and care paths. We will additionally audio record, code and analyse a subsample of 10 decision consultations per tumour type per hospital in both phases, to assess SDM (60 in both pre- and post-implementation, so 120 in total). In the post-implementation phase we will interview a subsample of 5 of the patients whose consultations were audiotaped per care path per country (30 in total) about their experiences with the redesigned care path.

Ad (2) and Ad (3): To evaluate the developed prognostic tools and conversation tools we will introduce the tools to both patients (in different stages of their illness) and healthcare professionals from four countries (UK, the Netherlands, Spain and Denmark). Twelve consultations in which the prognostic tool is used and 12 consultations in which the conversation tool is used will be audio-recorded. Qualitative, interpretative phenomenological analysis supported by conversation analysis techniques will be used to analyse the consultation encounter. Key themes will be generated and explorative comparisons between countries and settings will be made.

Ad (4): An online survey will be performed in Austria, Denmark, Germany, the Netherlands, Slovenia, Spain, Sweden and UK (*N* = 500 per country, total *N* = 4000) to study citizens’ experiences and attitudes regarding engagement in conversations about illness, cancer and palliative care, as well as the value of conversation tools, in particular the Metaphor Menu, which will be shown to survey participants.

Ad (5): To inform health technology and reimbursement bodies of different countries/legislations, short- and long-term health-economic consequences of MetroMapping will be assessed conducting a cost-effectiveness analysis (a) along the MetroMapping evaluation study using Bootstrap sampling and (b) in the case of melanoma using decision-analytic state-transition modelling^33,34^ for a lifelong time horizon. The model will be populated and validated with data derived from the pre-test and post-test studies, scores from the prognostic models and inpatient and outpatient costs, complemented by parameters from the published literature and national databases (cost catalogues, statistical life tables, etc.).

#### Work package 6: Policy (Objective 5, Months 1–60)

We will develop and evaluate MetroMapping and its embedded decision-support tools in three countries (Denmark, Spain and the Netherlands) and three cancer types (breast, prostate and melanoma). We also wish to explore the requirements for implementation of the MetroMapping methodology and the decision-support tools for patients with other types of cancer and in the other partner countries.

First, we will finalize the MetroMapping methodology in a Delphi consensus study with a total of 60 stakeholders in Denmark, Spain and the Netherlands. The group will work towards reaching consensus on the manual, to decide upon common elements as well as situation-specific elements, for international dissemination, resulting in an open resource manual, and building blocks for the metro line. We will build a business case for the implementation.

Next, we will evaluate the generalizability of the methodology and tools to other cancer types and other countries, in hospitals in Germany, Sweden and Slovenia, using qualitative interviews and focus groups with a total of 10–15 oncologists, patients and their significant others, service designers and quality of care staff per country (a total of approximately 40 interviews).

In each of the eight countries involved, we will next discuss the MetroMapping methodology, the decision-support tools and the study results in interviews with policymakers and guideline developers from ministries, health systems, hospitals, patient societies, oncology and palliative care professional societies, and payers, to facilitate implementation. We aim at 10–15 interviews per country, resulting in approximately 80–100 interviews.

#### Work Package 7: Ethics (Objective 6, Months 1–60)

The objective of this work package is to include social and ethical considerations in the entire development process of the data-driven digital support tools. We use an embedded ethics approach,^35,36^ supplemented with methods of empirical bioethics and bioethical ‘parallel research’.^37,38^ This aims to ensure that the models and tools are ethically and socially acceptable, truly enhance health, well-being and patient autonomy, do no unintended harm and contribute to health equity.

To achieve this, ethicists will be embedded in the project from the start. They will work closely together with researchers from the other work packages to anticipate, identify and address the ethical and societal issues that arise during the research, development and implementation trajectories. Embedded ethicists will regularly join meetings of the other work packages, pay visits to research teams and organize ethics meetings to learn about the work being done and to discuss emerging ethical questions or concerns. Issues that are expected to come up include risk of bias, transparency and explainability of the text mining models; and effects on inclusivity, equity and autonomy of the developed tools. The aim is to collaborate with members of the work packages in addressing those issues. Literature reviews and qualitative interviews with developers and end-users will also be conducted to support the ethical analyses and to integrate the moral views of those involved in the process. Drawing on the wealth of scholarly work in bioethics and digital ethics we will translate and integrate key ethical values and principles to the context of the 4D PICTURE project.

#### Work Package 8: Dissemination (Months 1–60)

To achieve efficient dissemination and communication of project information and innovation activities to key stakeholders and to increase the impact of the project, different communication tools will be used. From the start of the project, the 4D PICTURE project website (https://4dpicture.eu/) will deliver freely accessible information on decision-support tools to cancer patients, their significant others, clinicians and the public. As the project continues, additional traditional and modern (social) media will be used to deliver the main messages to the audience, using, for instance, e-newsletters, podcasts, virtual seminars and policy and awareness reports. Project-related content will be shared consistently through all named channels. Scientific data and development will be made freely accessible to professionals through peer-reviewed journal articles. Further, the prognostic tools and the conversation tool will be made open access available to the international community. Our dissemination strategy will be an ongoing dialogue with potential users throughout the duration of the 4D PICTURE project.

## Expected scientific contribution

With the cross-national and multidisciplinary consortium, we expect to achieve novel gains for patients with cancer, healthcare providers, citizens in general and for policy. These gains relate to decision-support tools, based on state-of-the art statistical modelling, text mining and design, on insight into patients’ preferences and experiences. A system approach will be taken to implement these tools in hospital settings. We hope that the developed tools and their implementation will result in improvements in the care paths of patients with cancer. Embedded ethicists will take care that potential issues will be anticipated, timely identified and addressed. Table 1 gives a detailed description of the key expected scientific contributions of the 4D PICTURE project.

**Table 1.** Key expected scientific contributions of the 4D PICTURE project.

Key element of the 4D PICTURE project	Current	Scientific contribution by the 4D PICTURE project
Care paths of cancer patients	Not resilient; poorly integrated and coordinated; disempowering patients; not person centred	Developed through design thinking; resilient and sustainable; addressing the needs of patients and clinicians
Decision-support tools	Rarely used, even less so in vulnerable groups; poorly integrated in oncological care paths; difficult to fit in diverse situations	Developed to be integrated in diverse care paths; system approach to implementation; targeted to wide range of cancer patients and clinicians
Modelling	Poor predictions of outcome and of individual treatment benefit; limited set of outcomes; uncertainty of predictions neglected	Robust modelling approaches for outcome and treatment benefit; patient centred outcomes; disentangle and quantify uncertainty
Insight into patients’ preferences and experiences	Usually obtained through time intensive and small scale qualitative interviews	Developing novel text mining methods to systematically analyse ‘big data’ patient experiences (such as blogs and patient fora)
Citizens	Often not involved in science or only for dissemination; often no direct benefit from science	Citizen science approach to leverage talents and insights of citizens; conversation tool developed for citizens to address taboo on cancer conversations
Ethics	Ethical evaluation of tool developed is often lacking or done only at end of the project, when the design is already fixed	Novel ‘embedded ethics’ approach, to anticipate, identify and address the ethical and societal issues that arise during the project

## Conclusion

Improved care paths integrating comprehensive decision-support tools have the potential to empower patients, their significant others and healthcare providers in decision-making and improve outcomes. This project will strengthen health care at the system level by improving its resilience and efficiency.

## Supplemental Material

sj-docx-1-pcr-10.1177_26323524231225249 – Supplemental material for Improving shared decision-making about cancer treatment through design-based data-driven decision-support tools and redesigning care paths: an overview of the 4D PICTURE project Click here for additional data file. Supplemental material, sj-docx-1-pcr-10.1177_26323524231225249 for Improving shared decision-making about cancer treatment through design-based data-driven decision-support tools and redesigning care paths: an overview of the 4D PICTURE project by Judith A. C. Rietjens, Ingeborg Griffioen, Jorge Sierra-Pérez, Gaby Sroczynski, Uwe Siebert, Alena Buyx, Barbara Peric, Inge Marie Svane, Jasper B. P. Brands, Karina D. Steffensen, Carlos Romero Piqueras, Elham Hedayati, Maria M. Karsten, Norbert Couespel, Canan Akoglu, Roberto Pazo-Cid, Paul Rayson, Hester F. Lingsma, Maartje H. N. Schermer, Ewout W. Steyerberg, Sheila A. Payne, Ida J. Korfage and Anne M. Stiggelbout in Palliative Care and Social Practice

## Declarations

**Ethics approval and consent to participate:** Ethics approval for this project will be sought from the Medical Ethics Committees of the participating centres. The consent to participate is not applicable for this article.

**Consent for publication:** Not applicable.

**Availability of data and materials:** So far no data have been collected. All reasonable requests for the data used in the 4D PICTURE project as well as the materials that will be developed will be seriously considered.
