# Supplementary material for: Improving shared decision-making about cancer treatment through design-based data-driven decision-support tools and redesigning care paths: an overview of the 4D PICTURE project
Source: Palliat Care Soc Pract. 2024 Feb 12;18:26323524231225249. doi: 10.1177/26323524231225249 (PMC10863384; doi:10.1177/26323524231225249)
Supplement: sj-docx-1-pcr-10.1177_26323524231225249 – Supplemental material for Improving shared decision-making about cancer treatment through design-based data-driven decision-support tools and redesigning care paths: an overview of the 4D PICTURE project [file sj-docx-1-pcr-10.1177_26323524231225249.docx]

Appendix 1. **Overview deliverables 4D PICTURE project in chronological order**

| **WORK PACKAGE** | **DELIVERABLE NAME** |
| --- | --- |
| 1 | Reports of the Consortium Meetings (M5, M14, M26, M38, M50, M59) |
| 8 | Letter to describe launch of project website (M6) |
| 8 | Initial and final version of Plan of Dissemination and Exploitation of Results (M6, M54) |
| 1 | Quality assurance manual including data management plan (M6, M60) |
| 1 | Report on main results of questionnaire studies (M12) |
| 2 | Process report on analysable datasets (M12) |
| 4 | Manual for the in-person training and e-learnings for the MetroMapping methodology (M12) |
| 3 | Report describing themes, values, preferences and concerns summarising experiences of cancer patients to act as input for WP4 (M18) |
| 2 | Report on first version of prediction algorithms (M24) |
| 3 | New English version of the conversation tool "Metaphor Menu" (M24) |
| 1 | Mid-term and final report on the Project Community Engagement Board activities (M30, M60) |
| 4 | Method, manual and tools for MetroMapping further expanded with decision-support tools as developed in WP2 and WP3 in an international context, and published on the MetroMapping.org website (M36) |
| 4 | Two redesigned care paths per country (DK, ES, NL) and, based on these, 12 examples of personal care path navigators, coproduced by clinicians, who are trained in SDM, and patients (M36) |
| 5 | Study initiation package including registration number of the mixed methods WP5 study in an approved registry, final version of study protocol as approved by the ethics committees and regulatory and ethics approvals (M36) |
| 7 | Reports about main results of literature review (M12) and qualitative interview study (M42) |
| 5 | Midterm recruitment report for mixed-methods study (M46) |
| 2 | Report on final cross validated prediction algorithms, including uncertainty quantification (M48) |
| 3 | Conversation tool in three additional languages (Danish, Dutch, Spanish) M48) |
| 6 | Manuals for MetroMapping for designers and hospital managers in four languages (M50) |
| 2 | Report on decision-support tools M54) |
| 3 | Report on the definition of the novel, multidisciplinary methods with executable exemplars, including a description of text mining and citizen science methods along with Python Notebooks (with source code and embedded documentation) to show examples running with proxy data (equivalent data that can be shared publicly for illustrative purposes) (M54) |
| 5 | Report on the status of posting results for mixed-methods study M56) |
| 7 | Report on the series of workshops (M58) |
| 6 | An extended website MetroMapping.org, with a community covering at least 8 European countries |
| 6 | Guide with implementation strategies of the decision-support tools and the MetroMapping methodology for policymakers, for international use |
| 7 | Report on the key ethical issues identified and addressed within 4D PICTURE |
| 8 | 15-20 (at least) accepted/submitted international peer reviewed publications |
